# Quantifying intra-urban socio-economic and environmental vulnerability to extreme heat events in Johannesburg, South Africa

**DOI:** 10.21203/rs.3.rs-5638179/v1

**Published:** 2025-04-02

**Authors:** Craig John Parker, Craig Mahlasi, Tamara Rosemary Govindasamy, Lebohang Radebe, Nicholas Brian Brink, Madina Doumbia, Yao Etienne Kouakou, Christopher Jack, Matthew Chersich, Guéladio Cissé, Sibusisiwe Makhanya

## Abstract

Urban populations face increasing vulnerability to extreme heat events, particularly in rapidly urbanising Global South cities where environmental exposure intersects with socioeconomic inequality and limited healthcare access. This study quantifies heat vulnerability across Johannesburg, South Africa, by integrating high-resolution environmental data with socio-economic and health metrics across 135 urban wards. We examine how historical urban development patterns influence contemporary vulnerability distributions using principal component analysis and spatial statistics. Environmental indicators (Land Surface Temperature (LST), vegetation indices, and thermal field variance) were combined with socioeconomic and health variables (including indicators on crowded dwellings and healthcare access, self-reporting of chronic diseases) in a comprehensive vulnerability assessment. Principal Component Analysis revealed three primary dimensions explaining 56.6% (95% CI: 52.4–60.8%) of the total variance: urban heat exposure (31.5%), health status (12.8%), and socio-economic conditions (12.3%). Built-up areas showed weak but significant correlations with heat indices (ρ = 0.28, p < 0.01), while higher poverty levels demonstrated moderate positive correlations with LST (ρ = 0.41, p < 0.001). The spatial analysis identified significant clustering of vulnerability (Global Moran's I = 0.42, p < 0.001), with distinct high-vulnerability clusters in historically disadvantaged areas. Alexandra Township showed the highest vulnerability(HVI score: 0.87, LST: 29.8°C ± 0.4°C, NDVI: 0.08 ± 0.02), with factors characterising the high vulnerability in that area including limited healthcare access and extreme heat exposure. Northern suburbs formed a significant low-vulnerability cluster (Mean HVI = 0.23 ± 0.07, p < 0.001), benefiting from greater vegetation coverage and better healthcare access. These findings demonstrate how historical planning decisions continue to shape contemporary environmental health risks, with vulnerability concentrated in areas of limited healthcare access and high extreme heat exposure. Results suggest the need for targeted interventions that address both environmental and social dimensions of heat vulnerability, particularly focusing on expanding healthcare access in identified hotspots and implementing community-scale green infrastructure in high-risk areas. This study provides an evidence-based framework for prioritising heat-resilience initiatives in rapidly urbanising Global South cities while highlighting the importance of addressing historical inequities in urban adaptation planning.

*Urban Heat Vulnerability, Spatial Analysis, Healthcare Access, Environmental Justice, Climate Adaptation, Principal Component Analysis, Johannesburg, Environmental Health*

## Statements and Declarations

### Acknowledgement

This research was conducted as part of the HE$^2$AT and Health in Africa Transdisciplinary (HEAT) Center initiative, supported by the NIH Common Fund under Award Number U54 TW 012083. We acknowledge the valuable contributions of the HE$^2$AT Center Group, which includes researchers, collaborators, and supporting staff from partner institutions. Their collective efforts in data sharing, technical support, and capacity building have significantly advanced this research.

We also extend our gratitude to the data owners and contributors who shared their datasets, enabling this study to integrate environmental, socioeconomic, and health metrics. Special thanks go to the Gauteng City-Region Observatory (GCRO) for providing the Quality of Life Survey data and to the United States Geological Survey for Landsat 8 satellite imagery.

Finally, we are grateful for the administrative and technical support provided by the HE$^2$AT Center Steering Committee and partner institutions and the guidance and input from the Publications Group. This publication reflects the authors' views and not necessarily those of the NIH or other supporting organisations.

### Ethics Approval

This research was conducted with approval from the Wits Human Research Ethics Committee in Johannesburg (reference number 200606). The study is based on secondary data analysis of publicly available datasets and followed the United States Department of Health and Human Services regulations for protecting human research subjects (45 CFR 46). All data were anonymous and received as aggregated statistics over small areas and were processed in accordance with ethical research principles.

### Consent to Participate

Not applicable - this study used secondary data analysis of publicly available datasets and did not involve direct human participants.

### Consent to Publish

Not applicable - this manuscript does not contain any individual person's data in any form.

### Data Availability

The datasets analysed for this study are available from the following sources:

- Environmental metrics were derived from ERA5 reanalysis data and Landsat 8 satellite imagery (December-February 2020-2021), available from the United States Geological Survey Earth Explorer
- Socio-economic and health data were obtained from the Gauteng City-Region Observatory (GCRO) Quality of Life Survey 2020-2021, publicly available at Gcro
- Analysis scripts for comparing GCRO data between survey periods are available at https://github.com/Logic06183/GCRO
- Complete analysis scripts, processed data, and visualisation code are available at https://github.com/Logic06183/data_sources
- Additional analysis files and supplementary materials are available from the corresponding author upon request

### Author Contributions

**Conceptualisation:** Craig Parker, Craig Mahlasi, Tamara Govindasamy, Matthew Chersich and Sibusisiwe Makhanya conceived and designed the study.

**Data acquisition, analysis, and interpretation:** Craig Parker, Craig Mahlasi, Tamara Govindasamy, Lebohang Radebe, Nicholas Brian Brink, Madina Doumbia, and Sibusisiwe Makhanya conducted data collection, processing, and analysis.

**Writing and revision:** Craig Parker wrote the original draft. Nicholas Brian Brink, Lebohang Radebe, Matthew Chersich, Gueladio Cisse, and Etienne Kouakou provided critical reviews and revisions of the manuscript.

**Final approval:** All authors reviewed and approved the final manuscript.

### Competing Interests

**Financial interests:** Authors affiliated with Wits Planetary Health Research (Craig Parker, Lebohang Radebe, Nicholas Brian Brink, Matthew Chersich) hold indirect investments in the fossil fuel industry through their institutional pension funds. Additionally, their institution, the University of the Witwatersrand, invests in the fossil fuel industry through endowments and other financial reserves.

**Non-financial interests**: The authors declare they have no non-financial competing interests.

.

## 1.Introduction

Climate change is significantly reshaping urban life, with extreme heat events becoming more frequent and severe [1, 2]. Urban populations face increasing vulnerability to these events, with risks shaped by complex interactions between environmental exposure, socioeconomic conditions, and health status [3, 4]. This vulnerability is particularly acute in rapidly urbanising Global South cities, where historical inequalities and limited adaptive capacity compound environmental challenges[5, 6].

Johannesburg, South Africa's largest city with 6.1 million inhabitants, presents a compelling case study of urban heat vulnerability[7]. The city's rapid urbanisation, pronounced socio-economic inequalities, and historical legacy of apartheid urban planning create distinct patterns of environmental risk[8, 9]. These factors interact with the urban heat island effect to produce heterogeneous vulnerability landscapes, particularly affecting disadvantaged populations [10]. Understanding these patterns is crucial for developing effective adaptation strategies, yet comprehensive analyses of urban heat vulnerability in African cities remain limited[2].

### 1.1. Historical Heat Events and Health Impacts in Johannesburg

Johannesburg has experienced increasingly frequent and intense heat events in recent decades. The city reached its highest recorded temperature of 38°C in January 2016 amid a severe heatwave and drought, breaking the previous record of 36.5°C just weeks earlier in November 2015[11]. These extreme temperatures have tangible health consequences. Studies in South Africa's major cities, including Johannesburg, show that above a city-specific threshold (~18.7°C apparent temperature for Johannesburg), all-cause mortality rises by ~0.9% per 1°C increase, and by 2.1% per 1°C among seniors 65+[12]. Adverse heat-related health events and excess mortality often concentrate in the elderly, while morbidity increases across vulnerable groups[13]. Hospitals report more patients for dehydration, renal failure, heat stroke, and other heat-induced illnesses during extreme heat spells. Although official records counted only 11 direct heat-related deaths in South Africa in one recent assessment, researchers estimate the actual heat mortality burden to be much higher when considering excess deaths, suggesting significant underreporting of heat-related health impacts[14].

### 1.2. Climate Change Projections for Johannesburg

Looking ahead, climate models project substantial warming for Johannesburg's region. Being an inland highland city, Johannesburg is expected to warm faster than global averages. By late-century (around 2100) under a high-emissions scenario, mean annual temperatures in interior South Africa could rise 6–7°C above late 20th-century baselines, compared to a 3–4°C increase in coastal areas[12]. Even mid-century projections are concerning: if global emissions remain high (RCP8.5), much of Johannesburg may warm by ~2°C by 2050, dramatically increasing heat extremes[12]. One projection shows that the number of hot nights (minimum temperature >20°C) in Johannesburg will currently quadruple from about 10 per year to ~40 per year by 2050 – and up to 100 hot nights in the city's most heat-prone neighborhoods[15]. Likewise, days over critical heat thresholds will become far more frequent. Researchers estimate an additional 3–4 weeks of very hot days per year in Johannesburg by mid-century, straining health systems and infrastructure[12, 16]. These projected temperature increases, especially under unabated climate change, imply a greater risk of heat-related illness and mortality[17]. The IPCC warns that beyond +2°C of global warming, heat-attributable mortality and morbidity in Africa will sharply rise[18]. In Gauteng, this could translate to a doubling of heat-related deaths without adaptation by the end of the century[19].

### 1.3. Apartheid Urban Planning and Current Vulnerability Patterns

Johannesburg's socio-spatial layout – largely a legacy of apartheid-era planning – is pivotal in shaping heat vulnerability today. Historically, apartheid policies created wealthy, low-density suburbs with ample green spaces (often reserved for white residents) and dense townships with minimal vegetation or services for non-white residents[20]. This has resulted in stark contrasts in urban heat exposure. Lush, tree-lined neighbourhoods like parts of Sandton enjoy cooler microclimates, while nearby townships such as Alexandra, with crowded tin-roof homes and few trees, register far higher temperatures[21]. A recent citizen-mapping campaign confirmed that Johannesburg's hottest areas are overwhelmingly in formerly segregated townships, which can be approximately 6°C hotter than the surrounding countryside on summer days (versus a 3–4°C urban heat island in greener suburbs)[15, 21, 22]. The lack of tree canopy and abundance of heat-absorbing surfaces in these areas directly stem from past planning inequities[15].

Moreover, housing quality differences exacerbate indoor heat exposure: informal housing, predominantly found in townships in South Africa (e.g., corrugated iron shacks), can become literal "ovens," with indoor temperatures up to 15°C higher than in modern housing during the day[23]. Residents of these neighbourhoods thus face a double burden – higher outdoor temperatures and more indoor heat retention – making them highly vulnerable during heatwaves. Apartheid geography has effectively hard-wired vulnerability into Johannesburg's landscape, clustering heat risk in the same marginalised communities that face economic and service deficits [23]. This explains why heat mortality and illness disproportionately affect poor, black communities in the city [24]

#### 1.4. Conceptual Framework and Current Knowledge

Urban heat vulnerability encompasses three interconnected dimensions: exposure, sensitivity, and adaptive capacity [1]. Exposure refers to the degree and duration of heat stress, typically quantified through temperature and environmental-related metrics and indices such as ambient air temperature above certain thresholds, Land Surface Temperature (LST), Urban Thermal Field Variance Index (UTFVI) and various human thermal discomfort indices. Heat metrics derived by integrating high spatial resolution satellite imagery and ground-based weather measurements provide high-resolution data on urban heat distribution [25]. Notably, dense urban areas with limited vegetation show significantly higher surface temperatures, with differences of up to 5°C compared to well-vegetated neighbourhoods[26-30]

Sensitivity reflects population susceptibility to heat stress, influenced by socio-economic conditions and health status. Recent studies demonstrate that chronic conditions such as cardiovascular disease, diabetes, and respiratory ailments significantly increase heat-related health risks[31-33]. These health vulnerabilities intersect with socio-economic disparities in Johannesburg, compounding heat-health risks in disadvantaged communities [7, 34].

Adaptive capacity, coping with and recovering from heat stress, depends heavily on access to healthcare, cooling infrastructure, and social support systems [35]. Limited healthcare access particularly affects heat vulnerability, as demonstrated by increased heat-related mortality in areas with restricted medical services [36]. In Johannesburg, historical planning decisions continue to influence the distribution of these adaptive resources, creating persistent spatial patterns of vulnerability [7].

#### 1.5. Previous Approaches to Heat Vulnerability Assessment

Heat Vulnerability Indices (HVIs) have emerged as essential tools for identifying at-risk populations from extreme heat events. The development of HVIs traces back to early work by Cutter et al. on social vulnerability mapping and was further refined by Reid et al., who created one of the first national heat vulnerability indices in the United States[37, 38]. Since then, three primary approaches to creating HVIs have emerged: equal-weighting methods where all variables contribute equally to the index; expert-weighting approaches where variables are weighted based on expert judgment; and statistical methods like Principal Component Analysis (PCA) that reduce dimensionality while objectively weighting variables based on their contribution to overall variance[39-41].

While HVIs have been widely applied in North America and Europe, comprehensive vulnerability assessments for African cities remain limited. Existing studies in Global South contexts have typically focused on environmental exposure (using satellite-derived temperature data) or social vulnerability, with few attempts to integrate both dimensions and health metrics[42]. This gap is particularly significant given the unique vulnerability contexts of rapidly urbanising African cities, where historical inequalities, informal settlements, and limited adaptive capacity create distinct risk profiles.

Our PCA approach offers several advantages for the Johannesburg context: it reduces subjectivity in variable weighting, identifies latent vulnerability dimensions that may not be immediately apparent, and allows for integrating diverse data types (environmental, socioeconomic, and health) into a unified framework. This study builds on previous HVI work by combining high-resolution environmental data with ward-level socioeconomic and health metrics to create a spatially explicit vulnerability assessment at a scale relevant to municipal planning and intervention.

#### 1.6. Research Gaps and Study Objectives

While existing research has examined individual components of heat vulnerability, three critical gaps remain:

Limited integration of environmental, socio-economic, and health data in vulnerability assessments, particularly in Global South contexts
Insufficient understanding of how historical urban development patterns influence contemporary heat vulnerability
Lack of high-resolution spatial analyses of heat vulnerability that can inform targeted intervention strategies

This study addresses these gaps through three specific objectives:

- Quantify the spatial distribution of heat vulnerability across Johannesburg by integrating high-resolution environmental data with socio-economic and health metrics

Analyse the relationship between historical urban development patterns and contemporary heat vulnerability

- Identify priority areas for intervention based on compound vulnerability factors

## 2.Data and Methods

### 2.1. Environmental Data Collection and Processing

We integrated environmental, socio-economic, and health data to assess heat vulnerability across Johannesburg's 135 wards. Environmental variables were derived from Landsat 8 Level 2, Collection 2 surface reflectance satellite imagery over path 170 and row 78 for the December-February 2020-2021 period. A maximum cloud cover of <10% was specified [24]. The data come readily corrected for atmospheric interference and calibrated to surface reflectance values. This selection yielded approximately one image per 16-day Landsat cycle, providing adequate temporal coverage while maintaining data quality. The thermal bands (10 and 11) were used to calculate LST using the split-window algorithm, which accounts for atmospheric effects by utilising the differential absorption between adjacent thermal infrared bands. This method, developed by Jiménez-Muñoz et al. (2014), incorporates atmospheric water vapour content derived from MODIS atmospheric profiles as an ancillary dataset to improve accuracy[43]. Surface reflectance calibration had already been applied to the Collection 2 Level 2 data, eliminating the need for additional atmospheric correction for the optical bands.

Each derived index served a specific purpose in our assessment framework: LST provided a direct measure of surface heating, NDVI quantified vegetation density (a key mediating factor for urban heat), UTFVI measured relative heat intensity compared to the city average, and NDBI identified densely built-up areas prone to heat accumulation. All processing was performed using the Google Earth Engine platform with standardised algorithms for consistent analysis across the study area[44].

We acknowledge that LST has limitations as a proxy for human heat exposure. As noted by Muse et al. (2024), LST represents the temperature of urban surfaces (roads, roofs, vegetation) as observed from satellite overpass and does not directly measure the air temperature that people experience. The relationship between LST and actual surface air temperature (SAT) can vary by season, weather conditions, and urban context. While LST provides valuable information on relative spatial patterns of urban heat, it may not capture absolute exposure or the full diurnal cycle of heat. We justify our use of LST due to its high spatial resolution and ability to identify neighbourhood-scale thermal variations, which are crucial for our intra-urban vulnerability analysis while recognizing that it represents one component of heat exposure rather than a comprehensive measure.

### 2.2. Urban Thermal Field Variance Index (UTFVI)

The Urban Thermal Field Variance Index (UTFVI) is a normalised measure that quantifies the intensity of urban heat island effects. It is calculated using the formula:

$$UTFVI = (Ts - Tmean)/Tmean$$

Where Ts is the LST at a specific location, and Tmean is the mean LST of the entire study area. This standardised approach allows for comparisons of relative heat intensity across different urban contexts. In our analysis, UTFVI values above 0.015 indicate areas with strong heat island effects, which often correspond to degraded urban thermal environments.

UTFVI offers several advantages as an environmental indicator of heat vulnerability. First, it normalises absolute temperature values relative to the city's average, highlighting anomalously hot areas regardless of the baseline temperature. Second, it provides a standardised classification scheme for thermal environmental quality: values below 0 indicate excellent or good conditions, values between 0-0.005 indicate normal conditions, values between 0.005-0.010 indicate poor conditions, values between 0.010-0.015 indicate bad conditions and values above 0.015 indicate worse conditions[45]. Previous studies have associated these categories with ecological and human comfort evaluations[46] . In the Johannesburg context, UTFVI helps identify neighbourhoods experiencing disproportionate heat stress compared to the city average, which may indicate areas requiring prioritised intervention[47].

#### 2.3. LST as Pragmatic Choice for Capturing Intra-Urban Heat Variability

While our environmental assessment utilised high-resolution (30 m) Landsat 8 data for intra-urban analysis, we acknowledge the resolution limitations of auxiliary climate data sources. ERA5 reanalysis data, with its horizontal resolution of approximately 31 km (~0.25°), was used only to provide broader climatic context and to identify extreme heat days at the city scale, not for intra-ward heat variability analysis. Although ERA5-Land offers enhanced resolution (9 km) and has shown improved performance in representing land surface processes compared to standard ERA5 [48], even this resolution would be insufficient for capturing neighbourhood-scale thermal variations in a complex urban environment like Johannesburg[49]For this reason, our intra-urban heat exposure analysis relied primarily on the high-resolution Landsat data to derive the LST.

#### 2.4. Gauteng City-Region Observatory Quality of Life Survey

This study's socioeconomic and health data were derived from the Gauteng City-Region Observatory (GCRO) Quality of Life Survey 2020-2021[50]. The GCRO is an independent research organisation established through a partnership between the University of Johannesburg, the University of the Witwatersrand, and the Gauteng Provincial Government. The Quality of Life survey is conducted biennially and serves as the most comprehensive assessment of living conditions, socioeconomic status, and well-being in the Gauteng province.

The 2020-2021 survey (6th iteration) employed a stratified random sampling approach designed to be statistically representative at the ward level. It involved 13,616 in-person household interviews across Gauteng, with 8,215 specifically in Johannesburg. The survey aimed to achieve approximately 60 respondents per ward, with a minimum threshold of 40 valid responses required for inclusion in our analysis. This approach resulted in comprehensive coverage of 135 of Johannesburg's 137 wards, with two excluded due to insufficient sample size.

It is important to note that the 2020-2021 survey coincided with the COVID-19 pandemic, which caused significant socioeconomic disruptions in Gauteng. To assess the impact of the pandemic on our vulnerability indicators, we conducted a comparative analysis with the pre-pandemic GCRO 2017/18 survey (see Supplementary Table S1)[51]. This analysis revealed that while absolute values of indicators changed substantially (e.g., unemployment increased from 3.76% to 18.67%), the spatial patterns of healthcare access variables maintained strong consistency ($r>0.8$). Economic indicators showed weaker spatial correlations, but the overall patterns of relative vulnerability remained largely stable. These findings suggest that our vulnerability assessment captures both chronic structural inequalities and acute pandemic impacts, with healthcare access-related vulnerabilities proving particularly stable across time periods.

For each ward, we extracted 18 variables related to socioeconomic status, healthcare access, and health conditions. These ward-level aggregates were calculated using appropriate weighting to ensure representativeness, with confidence intervals typically ranging from ±3.5% to ±5.0% at the 95% confidence level. The survey's rigorous quality control process included data validation, consistency checks, and imputation for selected variables where appropriate[50].

### 2.5. Selection of Indicators that Characterise Heat Vulnerability

Our selection of indicators that characterise heat vulnerability followed established frameworks that conceptualize heat vulnerability as a function of exposure, sensitivity, and adaptive capacity (Intergovernmental Panel on Climate Change, 2022)[18]. Each indicator was chosen based on empirical evidence linking it to heat-related health outcomes or adaptive responses:

**Environmental exposure indicators:**

- Land Surface Temperature (LST): Directly measures the thermal environment and has been linked to heat-related morbidity and mortality [52]
- Normalized Difference Vegetation Index (NDVI): Quantifies vegetation coverage, which mitigates urban heat through evapotranspiration and shading [53]
- Urban Thermal Field Variance Index (UTFVI): Identifies areas experiencing disproportionate heat stress relative to the city average [45]
- Normalized Difference Built-up Index (NDBI): Captures urban density, which influences heat retention and nocturnal cooling rates [54]

**Sensitivity indicators:**

- Demographic composition: Age structure and population density affect heat vulnerability, with children and older adults at higher risk [55]
- Chronic health conditions: Pre-existing conditions like diabetes, hypertension, and heart disease significantly increase susceptibility to heat stress [56]
- Housing quality: Crowded dwellings (>2 persons per room) and informal housing materials reduce thermal comfort and increase indoor heat exposure [57]
- Economic vulnerability: Food insecurity and participation in school feeding programs as an indicator of households with limited financial resources for heat adaptation [58]

**Adaptive capacity indicators:**

- Healthcare access: Distance to healthcare facilities (>1km), reliance on public healthcare, and lack of medical insurance limit access to treatment during heat emergencies[59]
- Infrastructure access: Availability of basic services like piped water affects the ability to maintain hydration and cooling during heat events [60]

Healthcare accessibility and health indicators were both derived from the GCRO survey data[61]. Healthcare accessibility was evaluated using three specific measures: (1) Distance to Healthcare Facilities, defined as the proportion of households within 1 km of a healthcare facility; (2) Public Healthcare Utilization Rates, capturing the percentage of residents reliant on public healthcare facilities; and (3) Medical Insurance Coverage, measuring residents' financial accessibility to healthcare services through private insurance. For health status assessment, we extracted the proportion of households reporting chronic conditions (diabetes, hypertension, heart disease) and recent experiences with infectious diseases (HIV, tuberculosis, COVID-19). These complementary measures allowed us to analyse both healthcare access barriers and underlying health vulnerabilities that may compound heat risk. All health-related data was self-reported at the household level and then aggregated to ward level with appropriate statistical weighting, providing a comprehensive spatial picture of health-related vulnerability factors across Johannesburg.

This comprehensive set of indicators ensures that all major dimensions of heat vulnerability are captured, allowing for a nuanced assessment of how different factors combine to create compound vulnerability profiles across Johannesburg's diverse neighbourhoods.

#### 2.6. Analytical Methods

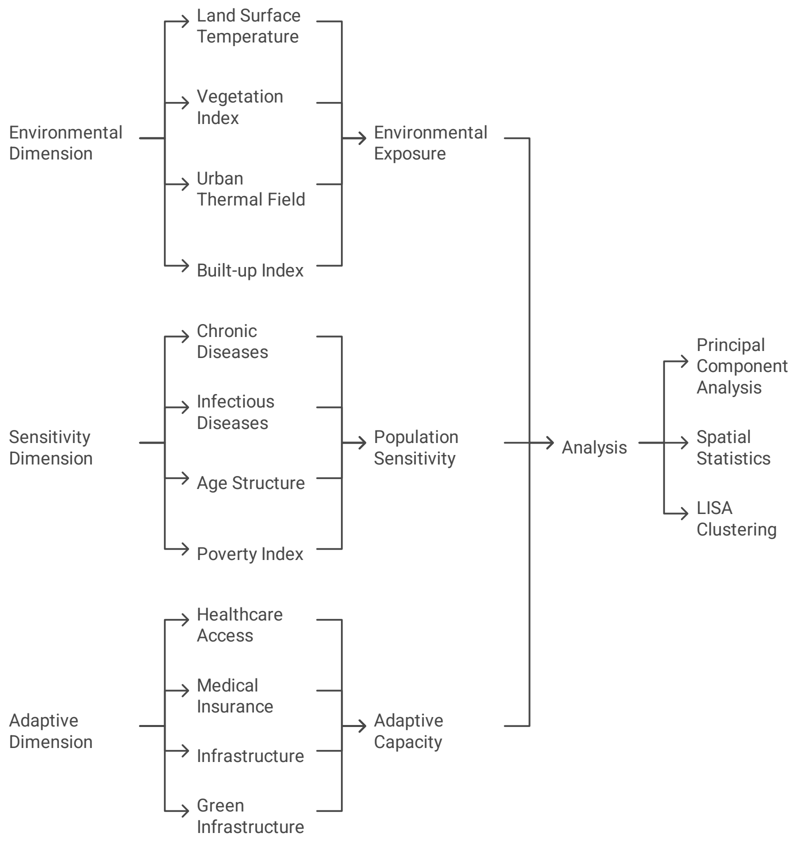

Figure 1: Analytical Framework for Assessing Urban Heat Vulnerability in Johannesburg: Integration of Environmental, Social, and Healthcare Dimensions. Schematic showing the methodological approach and data integration process.

We employed a three-stage analytical approach to assess heat vulnerability patterns across Johannesburg (Figure 1). First, we prepared the data by conducting an initial assessment of completeness (finding no missing values across the 22 variables), performing z-score normalisation to ensure comparability, and harmonising spatial data at the ward level with appropriate weighting schemes.

Before implementing Geographically Weighted Principal Component Analysis (GWPCA), we conducted preliminary analyses to ensure statistical appropriateness. This included correlation analysis to identify potentially redundant indicators (examining variables with $r > 0.9$ and retaining moderately correlated variables [$r = 0.6$-$0.9$] that represented conceptually distinct vulnerability aspects), and tests for normality using Shapiro-Wilk tests, which revealed non-normal distributions for most variables ($p < 0.05$). Given these non-normal distributions, we used Spearman correlation coefficients between all variable pairs ($\alpha = 0.05$) to avoid assumptions of linearity. The Kaiser-Meyer-Olkin measure of sampling adequacy (0.82) and Bartlett's test of sphericity ($p<0.001$) confirmed the appropriateness of our dataset for GWPCA despite correlations among variables.

The GWPCA, using optimal bandwidth selection through cross-validation, generated local eigenvalues and eigenvectors for each ward, with components retained based on eigenvalue >1 criterion. These components formed the basis of our Heat Vulnerability Index (HVI), weighted by explained variance and standardised to a 0-1 scale.

To address potential concerns regarding multicollinearity between LST and UTFVI (which is derived from LST), we conducted sensitivity analyses by creating three versions of our Heat Vulnerability Index: one with all variables (Original), one excluding LST (No_LST), and one excluding UTFVI (No_UTFVI). These sensitivity analyses helped verify the robustness of our vulnerability assessment approach (see Supplementary Materials, Section 9.3)

Spatial patterns were examined using Local Indicators of Spatial Association (LISA) analysis[62]. We constructed a queen contiguity weights matrix and calculated Local Moran's I values for the HVI, with significance assessed through 999 *Monte* Carlo permutations and p-values adjusted for multiple testing. This approach identified statistically significant ($p < 0.05$) high and low vulnerability clusters and spatial outliers. All analyses were conducted in R (version 4.1.2) using spdep (1.1-8) and GWmodel (2.2-8) packages, with visualisations created using ggplot2 and tmap. Analysis scripts are available in supplementary materials.

The LISA analysis classified areas into four types: high-high clusters (vulnerable areas surrounded by other vulnerable areas), low-low clusters (resilient areas surrounded by other resilient areas), and two types of spatial outliers - high-low (vulnerable areas surrounded by resilient areas) and low-high (resilient areas surrounded by vulnerable areas).

## 3.Results

### 3.1. Overview of Urban Heat Vulnerability Indicators

The initial analysis of the 135 wards revealed substantial variations in environmental, socioeconomic, and health indicators across Johannesburg (Table 1). Environmental metrics showed moderate variation, with LST ranging from 24.0°C to 30.7°C (mean = 27.9°C ± 1.3°C), while vegetation cover (NDVI) varied from 0.04 to 0.21, indicating significant differences in urban green space distribution.

Socioeconomic indicators demonstrated marked inequalities across wards. Access to basic infrastructure varied substantially: 94.5% of households across all wards had piped water, though in the most underserved wards, only 37.1% of households had access. Healthcare utilisation patterns showed similar disparities, with 63.47% (±30.49%) of households within each ward relying on public healthcare facilities city-wide. Food insecurity was a significant concern, with 32.8% of households at risk of hunger across all wards, rising to over 70% in the most vulnerable areas. Housing conditions also reflected these inequalities, with 15.2% of households living in crowded conditions city-wide, though this reached up to 84.6% in the most densely populated wards.

Health indicators revealed substantial variation in household health challenges across wards. Hypertension prevalence varied across regions, ranging from 15% in the healthiest wards to 47% in the most affected areas (mean = 23.00% ± 11.00%). Diabetes showed similar geographic variation, ranging from 7% to 35% across wards (mean = 11.00% ± 5.00%). During the study period, COVID-19 affected different areas of the city unequally, with prevalence ranging from 1% to 22% across wards (mean = 4.00% ± 4.00%). These self-reported health conditions suggest significant disparities in health vulnerabilities across the city's communities, reflecting the potential influence of multiple comorbidities and underlying risk factors that vary across populations.

### 3.2. Environmental-Social Correlations

Examination of Spearman rank correlations revealed several notable associations between socioeconomic indicators and environmental exposures (Table 5). Vegetation cover (NDVI) showed significant negative correlations with household overcrowding ($\rho=-0.56$, $p<0.001$) and food insecurity ($\rho=-0.58$, $p<0.001$), suggesting a potential protective effect of green space. Conversely, LST was positively correlated with crowding ($\rho$=0.29, p<0.01), lack of health insurance ($\rho$=0.30, p<0.01), and pneumonia self-reporting ($\rho$=0.26, p<0.01), indicating a convergence of heat exposure and social vulnerability.

Thermal field variance (UTFVI), a measure of extreme heat exposure, was negatively associated with participation in school feeding programs ($\rho$=-0.33, p<0.001). This suggests that higher heat stress areas may have unmet nutritional support needs. Strong intercorrelations were also observed among chronic health conditions, with pairwise Spearman coefficients ranging from 0.54 to 0.75 (p<0.001) for diabetes, hypertension, and heart disease.

#### 3.3. Component Analysis of Vulnerability Factors

Principal Component Analysis identified three significant components explaining 56.6% (95% CI: 52.4-60.8%) of total variance. The first component accounted for 31.5% of the variance (eigenvalue = 4.73), with the strongest loadings from environmental variables: UTFVI (0.35 ± 0.04), LST (0.34 ± 0.03), and negative loading from NDVI (-0.31 ± 0.03). The second component (12.8%) showed strongest loadings from health variables, while the third (12.3%) was dominated by socio-economic variables(Table 2).

Figure 2a presents the spatial distribution of the first principal component scores, which primarily captures environmental exposure factors. This component shows strong positive values in densely built areas with limited vegetation, particularly in the southern and northeastern regions. These areas are characterised by higher use of public healthcare, lower medical insurance coverage, higher household hunger risk, and higher LST. In contrast, negative scores (blue areas) represent more affluent regions with better environmental conditions that provide natural cooling and less heat stress, primarily in Johannesburg's central and northern parts.

The second component (Figure 2b), dominated by health variables, shows a somewhat different spatial pattern, with concentrations in areas of lower socioeconomic status but not perfectly aligned with environmental exposure patterns. Negative scores (blue areas) indicate wards with higher prevalence of chronic diseases, higher proportions of elderly population, and greater overall disease burden, creating populations that are physiologically more vulnerable to heat stress. This health vulnerability shows a more dispersed pattern than environmental factors, with pockets of high vulnerability scattered throughout the city, while northern areas generally show lower health vulnerability.

This spatial variability between components highlights how different dimensions of vulnerability have distinct geographic distributions, underscoring the importance of a multidimensional approach to vulnerability assessment. Wards that display both poor environmental conditions and vulnerable health populations represent the highest risk areas requiring prioritized interventions that address both dimensions simultaneously.

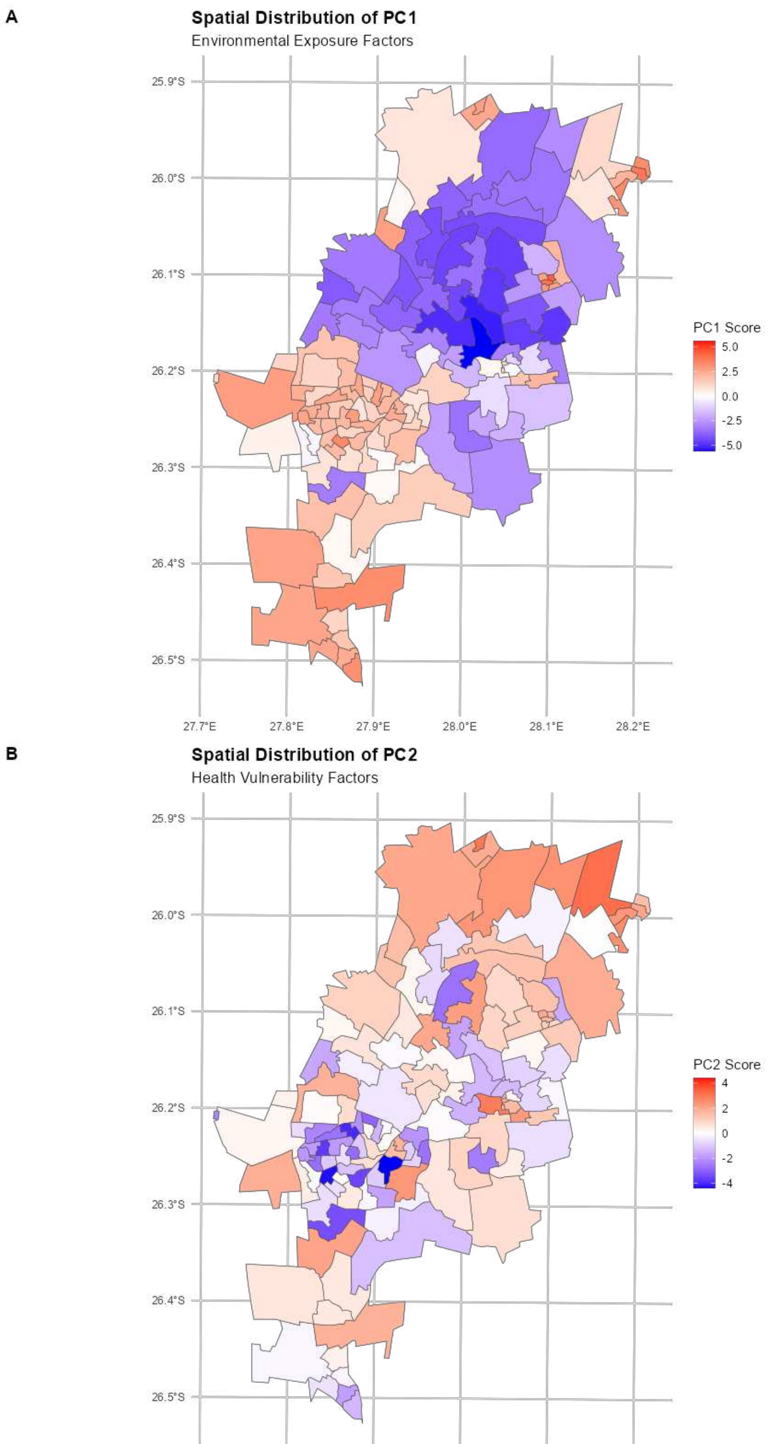

A
Spatial Distribution of PC1
Environmental Exposure Factors
25.9°S
26.0°S
26.1°S
26.2°S
26.3°S
26.4°S
26.5°S
27.7°E
27.8°E
27.9°E
28.0°E
28.1°E
28.2°E
PC1 Score
5.0
2.5
0.0
-2.5
-5.0
B
Spatial Distribution of PC2
Health Vulnerability Factors
25.9°S
26.0°S
26.1°S
26.2°S
26.3°S
26.4°S
26.5°S
PC2 Score
4
2
0
-2
-4

Figure 2: Spatial Analysis of Heat Vulnerability Components and Index in Johannesburg. (a) Spatial distribution of the first principal component scores representing environmental exposure factors, with higher values in densely built areas; (b) Spatial distribution of the second principal component scores representing health vulnerability factors.

We constructed a Heat Vulnerability Index (HVI) by combining these components, with each variable weighted according to its component loading and the proportion of variance explained by its respective component. The resulting index was standardised to a 0-1 scale. Figure 3a presents the spatial distribution of the HVI across Johannesburg, with darker colours indicating higher vulnerability. The map reveals a distinct north-south gradient in heat vulnerability, with concentrated areas of high vulnerability (orange) in the northeastern and southern regions of the city. A subset analysis of the ten most vulnerable wards (Figure 3b) highlights particular hotspots in Alexandra Township and parts of Soweto, where all three component factors (environmental exposure, health status, and socio-economic conditions) contribute to elevated vulnerability scores.

The HVI mapping reveals that the highest vulnerability scores (>0.75) are concentrated in historically disadvantaged areas, particularly those with limited healthcare access and high environmental exposure. In contrast, the northern suburbs consistently show lower vulnerability scores (<0.3), benefiting from greater vegetation cover and better healthcare infrastructure. This spatial pattern of vulnerability aligns with the component loadings from our PCA, demonstrating how environmental, health, and socio-economic factors combine to create distinct geographic risk patterns(Figure 2).

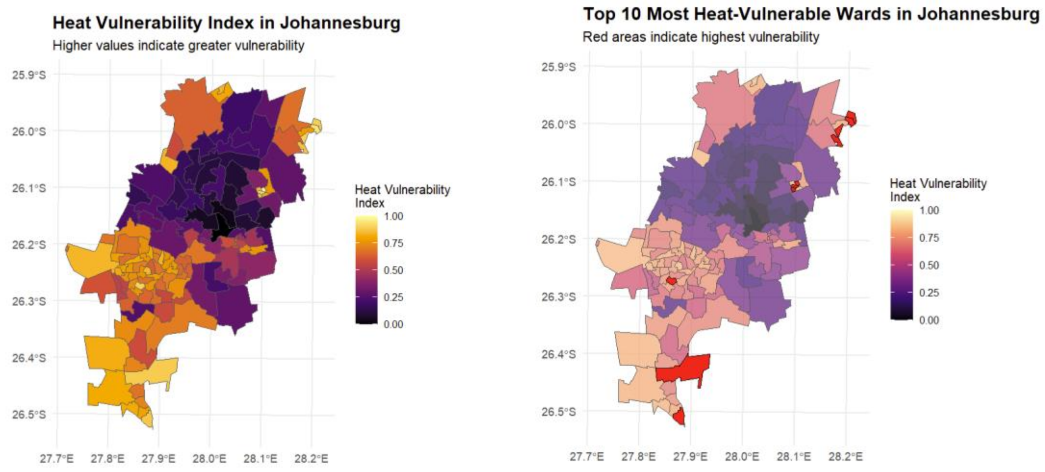

Figure 3(a) Composite Heat Vulnerability Index across all wards, with darker colors indicating higher vulnerability; (b) Detail of the ten most vulnerable wards, highlighting areas of concentrated risk. Ward boundaries are shown in black, and major roads are in grey.

#### 3.4. Spatial Distribution of Vulnerability

The LISA analysis revealed significant spatial clustering of heat vulnerability across Johannesburg (Global Moran's I = 0.42, $p < 0.001$). We identified 28 wards forming high-high clusters (Mean HVI = 0.78 ± 0.09, Moran's I = 0.68, $p < 0.001$), representing concentrated vulnerability hotspots. Conversely, 35 wards formed low-low clusters (Mean HVI = 0.23 ± 0.07, Moran's I = -0.55, $p < 0.001$), indicating areas of relative resilience. We also identified 20 spatial outliers, with 12 high-low outliers potentially indicating emerging hotspots and 8 low-high outliers suggesting localised resilience within otherwise vulnerable regions."

#### 3.5. Characteristics of High-Vulnerability Areas

Within these spatial patterns, five distinct high-vulnerability clusters emerged, each representing unique combinations of environmental, healthcare, and socio-economic challenges (Table 4)(Figure 4). Alexandra Township in the northeast emerged as the most vulnerable area, with extreme environmental exposure (LST: 29.8°C ± 0.4°C) combined with severely limited healthcare access. The Tembisa area showed intense urbanisation impacts (characterized by high built-up area index [NDBI > 0.40], low vegetation cover [NDVI < 0.10], and elevated land surface temperatureLST > 29°C]), while Tshepisong/Tshepiso demonstrated how infrastructure deficits (defined as >20% of households lacking access to one or more basic services including piped water, electricity, or proper sanitation) compound heat vulnerability. Notably, Lenasia South and Soweto clusters showed a different vulnerability pattern - while their LST readings were lower than other clusters (27.8°C and 27.4-29.6°C respectively), their vulnerability was driven by socioeconomic challenges including variable infrastructure quality and limited healthcare facility access, despite having relatively lower rates of public healthcare use and hunger risk compared to other high-vulnerability clusters. This variation in vulnerability drivers highlights the multidimensional nature of heat risk.

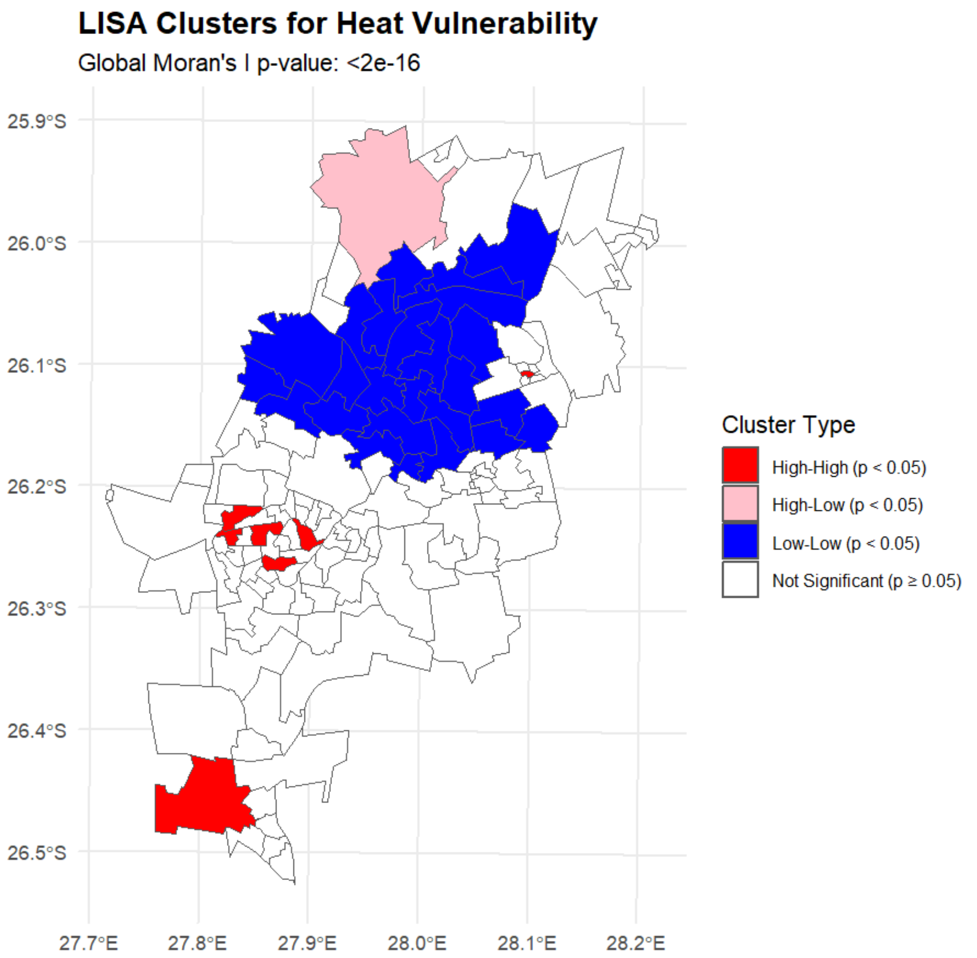

Figure 4: Spatial Clustering of Heat Vulnerability in Johannesburg Based on LISA Analysis. High-high clusters are shown in red, low-low clusters are shown in blue, and spatial outliers are shown in purple. Statistical significance at $p < 0.05$.

## 4. Discussion

### 4.2. Spatial Patterns of Heat Vulnerability

Our findings reveal how urban heat vulnerability in Johannesburg manifests through a complex interplay of environmental exposure, socio-economic conditions, and healthcare access. The stark spatial patterns we observed suggest that heat vulnerability is not merely a product of environmental factors but is deeply entwined with the city's historical development and ongoing socio-economic disparities.

The emergence of five distinct vulnerability clusters, each with its unique combination of risk factors, challenges conventional approaches to heat adaptation in Global South cities. In Alexandra Township, for instance, we found that extreme environmental exposure (LST: 29.8°C ± 0.4°C) coincides with severely limited healthcare access, with nearly 90% of residents dependent on distant public facilities. This coupling of environmental and social vulnerability creates a heightened vulnerability that exceeds what either factor would suggest in isolation. Similar patterns emerge in Tembisa and Tshepisong, where limited infrastructure and high population density amplify the effects of elevated surface temperatures.

Perhaps most striking is how these vulnerability patterns align with Johannesburg's historical spatial segregation. The strong statistical clustering we observed (Global Moran's I p-value: <2e-16) reveals that heat vulnerability follows clear socio-spatial lines, with historically disadvantaged areas showing significantly higher risk profiles. This finding extends previous work on environmental justice in South African cities by quantifying how historical planning decisions continue to shape contemporary environmental health risks[63].

The clear north-south divide in vulnerability patterns raises important questions about urban development and adaptation capacity. While northern suburbs benefit from abundant vegetation and robust healthcare infrastructure, creating significant low-vulnerability clusters, southern regions face compounded challenges. These lower vulnerability areas (defined quantitatively by HVI scores <0.25, vegetation cover above the city median, medical insurance coverage >60%, and household hunger risk <15%) represent neighbourhoods with both environmental buffers against heat and socioeconomic resources that enhance adaptive capacity. This disparity suggests that conventional approaches to heat adaptation, which often focus solely on environmental interventions like urban greening, may be insufficient without parallel investments in social infrastructure and healthcare access.

Our identification of spatial outliers - particularly high-vulnerability pockets within generally resilient areas - highlights the dynamic nature of urban heat risk. These anomalies often represent informal settlements or areas of rapid urban transformation, suggesting that vulnerability patterns are not static but evolve with urban development. This finding has important implications for adaptation planning, indicating the need for flexible, responsive interventions that can address emerging vulnerability hotspots.

#### 4.3. Environmental-Social Correlations and Underlying Mechanisms

The correlations observed in the analysis reflect complex socio-spatial relationships in Johannesburg's urban landscape. The negative correlation between vegetation cover and overcrowding stems from historical urban development patterns where higher-density, lower-income areas were developed with minimal green space planning. Similarly, the positive correlation between LST and lack of health insurance represents the spatial concentration of disadvantage in Johannesburg, where areas of higher environmental stress often coincide with lower socioeconomic status. These are not necessarily causal relationships but manifestations of how historical socio-spatial segregation has created overlapping environmental and social vulnerabilities in specific city areas. The correlation between UTFVI and school feeding programs may indicate that areas experiencing the most intense urban heat effects have socio-economic profiles different from areas with high absolute temperatures but less extreme heat island effects.

#### 4.4. Implications for Urban Planning and Heat Adaptation

These findings have significant implications for urban policy and planning. While targeted interventions in high-vulnerability clusters are needed, our results suggest that such interventions must simultaneously address multiple dimensions of vulnerability. Simple environmental modifications, while important, may only be sufficient with corresponding improvements in healthcare access and social infrastructure. The clear spatial patterns we observed provide a strong empirical basis for prioritising interventions while identifying compound risk factors, suggesting the need for integrated adaptation strategies.

Our study also reveals several important areas for future research. The strong spatial clustering we observed suggests the need for a more detailed investigation of neighbourhood-level adaptation mechanisms, particularly in high-vulnerability areas. Additionally, identifying spatial outliers raises questions about the dynamics of vulnerability development and the potential for preventive interventions. Future work might also explore how these vulnerability patterns might shift under different climate change scenarios.

These findings advance our understanding of urban heat vulnerability in several essential ways. First, they provide empirical evidence for the spatial clustering of heat risk in a major Global South city. Second, they demonstrate how historical planning decisions shape contemporary environmental health risks. Finally, they reveal the complex interactions between environmental exposure and social vulnerability that must be addressed in urban adaptation planning. As cities worldwide grapple with increasing heat stress under climate change, these insights offer valuable lessons for developing more equitable and effective adaptation strategies.

## 5. Limitations

### 5.1. Data Limitations

Although the GCRO Quality of Life Survey provides a rich dataset for exploring urban vulnerabilities, it has some constraints. The survey is not primarily designed as a health assessment, so the derived health metrics should be interpreted as general indicators rather than precise prevalence estimates. Additionally, the self-reported nature of the data may introduce biases, particularly for sensitive health topics.

### 5.2. Multicollinearity Assessment and Variable Sensitivity

A potential limitation is including both LST and UTFVI in our PCA, since UTFVI is derived from LST normalisation. We conducted sensitivity analyses with alternative HVI versions excluding either variable to address this. Results showed remarkable consistency (correlations >0.99, stable PC1 variance of 31.46%-32.02%, and identical top 10 vulnerable wards across all versions). This confirms our vulnerability assessment is robust despite the correlation between these variables (see Supplementary Materials, Section 9.3).

### 5.3. Spatial Scale Considerations

Another limitation concerns the spatial scale of our analysis. Using administrative wards as our unit of analysis provided practical advantages regarding data availability and policy relevance, but introduces potential modifiable areal unit problems (MAUP). Our analysis of local LST variability revealed that in most wards (94.8%), the local temperature variation was less than the global standard deviation across Johannesburg (1.33°C), supporting our ward-level approach. However, 18.5% of wards exhibited substantial local variability with standard deviations exceeding 1°C, with the highest reaching 2.03°C.

This pattern was more pronounced in larger wards, with 23.9% of above-median sized wards showing high local variability (correlation between ward size and variability: $r = 0.236$). These findings suggest that while our ward-level analysis is appropriate for most of Johannesburg, some larger peripheral wards may contain significant internal temperature gradients that could obscure localized vulnerability hotspots. Future research could employ finer-scale analysis using dasymetric mapping or grid-based approaches to address this limitation, particularly in identified hotspot areas.

#### 5.4. Generalizability and Future Research Directions

A notable contextual limitation is that data collection occurred during the COVID-19 pandemic. As detailed in section 2.4 and Supplementary Table S1, our comparative analysis with pre-pandemic data showed that while absolute values of socioeconomic indicators changed substantially, spatial patterns of healthcare vulnerability maintained strong consistency ($r>0.8$). This suggests our findings represent enduring structural patterns rather than solely pandemic effects, though acute pandemic impacts likely intensified existing vulnerabilities in the most affected communities.

Our focus on Johannesburg offers a detailed case study but may limit the generalizability of the findings to other urban contexts. Cities vary in their specific environmental challenges, socioeconomic conditions, and governance structures, so the patterns observed here may not be universally applicable. Comparative studies across multiple cities would help assess the transferability of our vulnerability assessment approach and identify common themes and context-specific differences. Finally, while we examined a broad set of vulnerability indicators, additional factors that influence heat risk are likely not captured in our analysis. For example, we did not have access to high-resolution data on housing quality, social capital, or behavioural adaptations, which could all shape resilience to heat stress. Integrating a wider range of data sources and vulnerability proxies could provide an even more comprehensive picture.

Despite these limitations, our study demonstrates the value of integrating environmental, socioeconomic, and health data to map urban vulnerability. Identifying key correlations and spatial patterns highlights potential intervention points and future research directions to enhance urban resilience. As cities grapple with the challenges posed by climate change, such interdisciplinary vulnerability assessments will be crucial for guiding equitable adaptation efforts.

### 6.Conclusions

This study provides a comprehensive spatial analysis of heat vulnerability in Johannesburg, revealing how environmental exposure and social inequality create distinct geographic risk patterns. Integrating multiple data streams and advanced spatial statistics, we identified five geographically distinct high-vulnerability clusters, each characterised by unique combinations of environmental exposure, healthcare limitations, and socioeconomic challenges (Global Moran's I p-value: $<2e-16$). These vulnerability patterns demonstrate how historical planning decisions shape contemporary environmental health challenges in South African cities.

Our findings challenge simplistic approaches to urban heat adaptation. While environmental factors account for the largest variance (31.5%), health access and socio-economic conditions play crucial mediating roles. While acknowledging potential within-ward variations, the spatial precision of our analysis, examining 135 wards across Johannesburg, provides actionable intelligence for urban planning and public health interventions. In identified hotspots like Alexandra

Township and Tembisa, extreme surface temperatures combined with severely limited healthcare access create a compound vulnerability that neither factor would predict

The strong correlations between environmental and socioeconomic factors reflect deep-rooted socio-spatial patterns requiring coordinated intervention strategies. As cities worldwide confront increasing heat stress due to climate change, our findings suggest that effective adaptation requires simultaneously addressing environmental exposure and social vulnerability. Future research should examine how these vulnerability patterns evolve and evaluate intervention effectiveness. Most crucially, our results indicate that climate change may exacerbate existing urban inequalities without concerted efforts to address both environmental and social dimensions of heat vulnerability.

### 7.Tables

Table 1. Summary of Environmental, Socioeconomic, and Health Indicators Across Johannesburg Wards (N=135)

| Environmental Indicators | Mean ± SD | Min | 25% | Median | 75% | Max |
|---|---|---|---|---|---|---|
| Land Surface Temperature (°C) | 27.92 ± 1.33 | 23.99 | 27.01 | 27.93 | 28.86 | 30.66 |
| UTFVI | -0.06 ± 0.04 | -0.18 | -0.08 | -0.06 | -0.03 | 0.02 |
| NDVI | 0.14 ± 0.04 | 0.04 | 0.11 | 0.14 | 0.17 | 0.21 |
| NDBI | 0.35 ± 0.03 | 0.34 | 0.36 | 0.37 | 0.42 | 0.43 |
| | | | | | | |
| Socioeconomic Indicators | Mean ± SD | Min | 25% | Median | 75% | Max |
| Crowded Dwellings (%) | 15.19 ± 11.97 | 0.00 | 9.49 | 15.01 | 23.73 | 84.62 |
| Without Piped Water (%) | 5.45 ± 9.45 | 0.00 | 0.00 | 1.89 | 5.67 | 62.95 |
| Using Public Healthcare (%) | 63.47 ± 30.49 | 3.56 | 31.75 | 73.69 | 87.80 | 98.55 |
| Without Medical Insurance (%) | 63.47 ± 26.44 | 7.07 | 38.85 | 73.59 | 84.62 | 97.11 |
| At Risk of Hunger (%) | 32.84 ± 21.91 | 0.00 | 9.49 | 36.78 | 49.73 | 70.01 |
| School Feeding Schemes (%) | 31.33 ± 21.32 | 0.00 | 10.49 | 33.72 | 49.32 | 79.63 |
| | | | | | | |
| Health Indicators | Mean ± SD | Min | 25% | Median | 75% | Max |
| Poor Health (%) | 7.18 ± 4.48 | 0.00 | 3.97 | 6.09 | 9.95 | 19.98 |
| Unable to Access Healthcare (%) | 4.20 ± 3.67 | 0.00 | 1.50 | 3.83 | 5.58 | 16.97 |

| | | | | | | |
|---|---|---|---|---|---|---|
| Diabetes (%) | 11.00 ± 5.00 | 0.00 | 7.00 | 11.00 | 15.00 | 35.00 |
| Heart Disease (%) | 5.00 ± 4.00 | 0.00 | 2.00 | 4.00 | 7.00 | 27.00 |
| Hypertension (%) | 23.00 ± 11.00 | 0.00 | 15.00 | 23.00 | 31.00 | 47.00 |
| HIV (%) | 8.00 ± 7.00 | 0.00 | 3.00 | 7.00 | 12.00 | 27.00 |
| TB (%) | 3.00 ± 4.00 | 0.00 | 0.00 | 2.00 | 5.00 | 12.00 |
| COVID-19 (%) | 4.00 ± 4.00 | 0.00 | 1.00 | 3.00 | 6.00 | 22.00 |

Note: UTFVI = Urban Thermal Field Variance Index; NDVI = Normalized Difference Vegetation Index; NDBI = Normalized Difference Built-up Index. Values represent mean ± standard deviation or percentage where applicable.
For Using Public Healthcare (%), standard deviation reflects the high variability between wards (30.49%) due to clustered access patterns. Twenty-five percent (25%) and seventy-five percent (75%) percentiles are shown as 25% and 75% respectively

Table 2. Principal Component Analysis Results for Heat Vulnerability Indicators

| Component | Eigenvalue | Variance Explained (%) | Cumulative Variance (%) | Key Contributing Variables (Loading > 0.3) |
|---|---|---|---|---|
| PC1 (Urban Heat Exposure) | 4.73 | 31.5 | 31.5 | UTFVI (0.35), LST (0.34), NDBI (0.32), NDVI (-0.31) |
| PC2 (Health Status) | 1.92 | 12.8 | 44.3 | Chronic Diseases (-0.32), COVID-19 (-0.30), Healthcare Use (-0.22) |
| PC3 (Socio-economic) | 1.85 | 12.3 | 56.6 | Crowded Dwellings (-0.30), Household Hunger (-0.28) |

Note: Only loadings > |0.2| shown. Kaiser-Meyer-Olkin (KMO) measure = 0.82, Bartlett's test p < 0.001. PC = Principal Component

Table 3. LISA Cluster Analysis Summary

| Cluster Type | N (Wards) | Mean HVI | Moran's I | p-value | Key Characteristics |
|---|---|---|---|---|---|
| High-High | 28 | 0.78 ± 0.09 | 0.68 | <0.001 | LST > 29°C, Limited Healthcare |
| Low-Low | 35 | 0.23 ± 0.07 | -0.55 | <0.001 | High Vegetation, Good Healthcare |
| High-Low | 12 | 0.65 ± 0.11 | 0.32 | <0.05 | Informal Settlements |
| Low-High | 8 | 0.35 ± 0.08 | -0.28 | <0.05 | Green Space Islands |

Note: HVI = Heat Vulnerability Index (0-1 scale), ± indicates standard deviation. All spatial statistics calculated using queen contiguity weights matrix.

Table 4. Characteristics of High-Vulnerability Areas

| Region | Environmental Indicators | Healthcare Access | Socio-Economic Factors | Health Indicators |
|---|---|---|---|---|
| Alexandra Township | LST: 29.8°C ± 0.4°C; NDVI: 0.08 ± 0.02 | Public healthcare use: 89.2%; Avg distance to facilities: >3km | Household hunger risk: 70.0%; Crowded dwellings: 37.5% | Hypertension rate: 1.5× avg; Chronic disease burden: High |
| Tembisa Areas | LST: 28.9°C ± 0.5°C; NDVI: 0.13 ± 0.03 | Public healthcare use: 78.5%; Avg distance to facilities: 2.8km | Household hunger risk: 56.4%; Crowded dwellings: 32.5% | COVID-19 rates: Above avg; TB prevalence: High |
| Tshepisong/Tshepiso | LST: 29.2°C ± 0.3°C; NDVI: 0.11 ± 0.03 | Public healthcare use: 67.8%; Avg distance to facilities: 3.1km | Household hunger risk: 58.4%; No piped water: 45.2% | Infectious disease: High; Healthcare access: Limited |
| Lenasia South | LST: 27.8°C ± 0.6°C; NDVI: 0.14-0.22 | Public healthcare use: 64.5%; Avg distance to facilities: 2.5km | Household hunger risk: 52.3%; Crowded dwellings: 26.8% | Chronic disease: High; Healthcare access: Limited |
| Soweto Clusters | LST: 27.4-29.6°C; NDVI: 0.09-0.16 | Public healthcare use: 75.8%; Avg distance to facilities: 2.9km | Household hunger risk: 48.2%; Crowded dwellings: 28.4% | Chronic disease: Above avg; Healthcare access: Variable |

Note: LST = Land Surface Temperature; NDVI = Normalized Difference Vegetation Index. All metrics shown with standard deviations where applicable.

**Table 5: Correlation Matrix of Key Variables (Spearman's ρ)**

| Variable | LST | NDVI | Healthcare Access | Poverty Index |
|---|---|---|---|---|
| LST | 1*** | -0.29*** | 0.28** | 0.41*** |
| NDVI | -0.29*** | 1*** | -0.65*** | -0.56*** |
| Healthcare Access | 0.28** | -0.65*** | 1*** | 0.83*** |
| Poverty Index | 0.41*** | -0.56*** | 0.83*** | 1*** |

Note: Significance levels *** $p < 0.001$, ** $p < 0.01$, * $p < 0.05$. N = 135 wards. LST = Land Surface Temperature; NDVI = Normalized Difference Vegetation Index.

### 8.References

1. IPCC Working Group, I., *Climate Change 2022: Impacts, Adaptation and Vulnerability. Contribution of Working Group II to the Sixth Assessment Report of the Intergovernmental Panel on Climate Change*. 2022, Cambridge University Press.
2. Ansah, E.W., et al., *Climate change, urban vulnerabilities and adaptation in Africa: a scoping review.* Climatic Change, 2024. **177**(4): p. 71.
3. Estoque, R.C., Ooba, M., Avitabile, V., Hijioka, Y., DasGupta, R., Togawa, T., Murayama, Y., Nakamura, S., & Sun, Y., *The future of Southeast Asia's forests.* Nature Communications, 2023. **10**(1): p. 1829-1829.
4. Tuholske, C., Caylor, K., Funk, C., Verdin, A., Sweeney, S., Grace, K., Peterson, P., & Evans, T., *Global urban population exposure to extreme heat.* Proceedings of the National Academy of Sciences, 2021. **118**(41): p. e2024792118-e2024792118.
5. Tietjen, B., Jacobsen, K., & Hollander, J., *Climate Change and Urban Migration in Sub-Saharan African Cities: Impacts and Governance Challenges.* Journal of Climate Resilience and Justice, 2023. **1**: p. 20-32.
6. Abrahams, D.E.D., *City Scoping Study: Johannesburg.* African Centre for Cities, 2019.
7. Souverijns, N., De Ridder, K., & Veldeman, N., *Urban heat in Johannesburg and Ekurhuleni, South Africa: A meter-scale assessment and vulnerability analysis.* Urban Climate, 2022. **46**: p. 101331.
8. Giombini, V. and J.P.R. Thorn, *Urban Green Spaces in a Post-Apartheid City: Challenges and Opportunities for Nature-based Solutions*, in *Human-Nature Interactions: Exploring Nature's Values Across Landscapes*, I. Misiune, D. Depellegrin, and L. Egarter Vigl, Editors. 2022, Springer International Publishing: Cham. p. 207-219.
9. Statistics South, A., *Urban Development Report 2023.* Statistics South Africa, 2023.
10. Schlosberg, D.R.L.B.J., *Environmental justice and attachment to place: Australian cases.* Environmental Politics, 2022. **31**(1): p. 1-20.
11. Strydom, N., *Johannesburg temperature hits record high as South Africa drought persists*, in *Reuters*. 2016.
12. Wichmann, J., *Heat effects of ambient apparent temperature on all-cause mortality in Cape Town, Durban and Johannesburg, South Africa (2006–2010).* Science of The Total Environment, 2017. **587-588**: p. 266-272.
13. Arsad, F.S., et al., *The Impact of Heatwaves on Mortality and Morbidity and the Associated Vulnerability Factors: A Systematic Review.* International Journal of Environmental Research and Public Health, 2022. **19**(23): p. 16356.
14. Scovronick, N., Sera, F., Acquaotta, F., Garzena, D., & Gasparrini, A. (2018), *The association between ambient temperature and mortality in South Africa: A time-series analysis.* Environmental Research, 2018. **161**: p. 229-235.
15. World Bank Cities Support, P., *Heat mapping by citizen scientists points the way to a cooler future*, in *Business Day (11 March 2024)*. 2024.
16. Engelbrecht, F., Adegoke, J., Bopape, M.J., Naidoo, M., Garland, R., Thatcher, M., McGregor, J., Katzfey, J., Werner, M., Ichoku, C. and Gatebe, C.,, *Projections of rapidly rising heat extremes in South Africa.* Journal of Climate, 2015.
17. Chersich, M., *"Africa is getting hotter" – Wits University News.* Wits University, 2023.

18. IPCC Working Group, I., *Climate Change 2022: Impacts, Adaptation and Vulnerability – Africa Chapter*, in *Intergovernmental Panel on Climate Change*. 2022.
19. Garland, R.M. and et al., *Estimating future heat-related mortality in Gauteng*. Global Health Action, 2015. **7**(1): p. 26713.
20. Giombini, V., & Thorn, J. P. R., *Urban Green Spaces in a Post-Apartheid City: Challenges and Opportunities for Nature-Based Solutions.* Human-Nature Interactions: Exploring Nature's Values Across Landscapes, 2022. **1**: p. 207-219.
21. Souverijns, N., De Ridder, K., & Veldeman, N., *Urban heat in Johannesburg and Ekurhuleni, South Africa: A meter-scale assessment and vulnerability analysis.* Urban Climate, 2023. **47**: p. 101331.
22. Habitat, U.N., *World Cities Report 2024 – Box 4.2: Johannesburg's Urban Heat Rise*, in *United Nations Human Settlements Programme*. 2023.
23. Naicker, N., et al., *Indoor Temperatures in Low-Cost Housing in Johannesburg, South Africa.* International Journal of Environmental Research and Public Health, 2017. **14**(11): p. 1410.
24. Giombini, V. and J.P.R. Thorn, *Urban Green Spaces in a Post-Apartheid City: Challenges and Opportunities for Nature-based Solutions*, in *Human-Nature Interactions: Exploring Nature's Values Across Landscapes*, I. Misiune, D. Depellegrin, and L. Egarter Vigl, Editors. 2022, Springer: Cham. p. 207-219.
25. Wang, Y.U.B.R., *Urban resilience to climate change: A systematic literature review.* Sustainable Cities and Society, 2023. **85**: p. 104080-104080.
26. Li, X., Zhou, Y., Asrar, G. R., Imhoff, M., & Li, X., *The surface urban heat island response to urban expansion: A panel analysis for the conterminous United States.* Science of The Total Environment, 2017. **605-606**: p. 426-435.
27. Oke, T.R., *The energetic basis of the urban heat island.* Quarterly journal of the royal meteorological society, 1982. **108**(455): p. 1-24.
28. Santamouris, M., *Analyzing the heat island magnitude and characteristics in one hundred Asian and Australian cities and regions.* Science of The Total Environment, 2015. **512-513**: p. 582-598.
29. Voogt, J.A. and T.R. Oke, *Thermal remote sensing of urban climates.* Remote sensing of environment, 2003. **86**(3): p. 370-384.
30. Zhou, D., Zhao, S., Liu, S., Zhang, L., & Zhu, C., *Surface urban heat island in China's 32 major cities: Spatial patterns and drivers.* Remote Sensing of Environment, 2014. **152**: p. 51-61.
31. Watts, N.A.M.A.N. and et al., *The 2023 report of the Lancet Countdown on health and climate change: health at the mercy of fossil fuels.* The Lancet, 2023. **402**(10397): p. 1610-1654.
32. Khosla, R., A. Jani, and R. Perera, *Health risks of extreme heat.* BMJ, 2021. **375**: p. n2438.
33. Desai, Y., H. Khraishah, and B. Alahmad, *Heat and the Heart.* Yale J Biol Med, 2023. **96**(2): p. 197-203.
34. Urban Heat Study, G., *Urban Heat Islands in South African Cities: A Comprehensive Analysis.* South African Environmental Observation Network, 2023.

35. World Health, O., *Global Report on Urban Health: Equitable, Healthier Cities for Sustainable Development.* World Health Organization, 2023.
36. Ansah, E.W., et al., *Health systems response to climate change adaptation: a scoping review of global evidence.* BMC Public Health, 2024. **24**(1): p. 2015.
37. Cutter, S.L., B.J. Boruff, and W.L. Shirley, *Social Vulnerability to Environmental Hazards.* Social Science Quarterly, 2003. **84**(2): p. 242-261.
38. Reid, C.E., et al., *Mapping community determinants of heat vulnerability.* Environmental Health Perspectives, 2009. **117**(11): p. 1730-1736.
39. Chow, W.T.L., W.-C. Chuang, and P. Gober, *Vulnerability to extreme heat in metropolitan Phoenix: spatial, temporal, and demographic dimensions.* The Professional Geographer, 2012. **64**(2): p. 286-302.
40. Wolf, T. and G. McGregor, *The development of a heat wave vulnerability index for London, UK.* Weather and Climate Extremes, 2013. **1**: p. 59-68.
41. Zhu, Q., Liu, T., Lin, H., Xiao, J., Luo, Y., Zeng, W., Zeng, S., Wei, Y., Chu, C., Baum, S., Du, Y., & Ma, W, *Spatial distribution of health vulnerability to heat waves in Guangdong, China.* Global Health Action, 2014. **7**: p. 25051.
42. Niu, S. and et al., *A systematic review of heat vulnerability index development and validation.* Environmental Research Letters, 2021. **16**(5): p. 053007.
43. Jiménez-Muñoz, J.C., et al., *Land surface temperature retrieval methods from Landsat-8 thermal infrared sensor data.* IEEE Geoscience and Remote Sensing Letters, 2014. **11**(10): p. 1840-1843.
44. Google Earth, E. and I. Landsat 8 Satellite, *Environmental Indices for Johannesburg: LST, NDVI, and NDBI Calculated in Google Earth Engine*. 2021.
45. Zhang, Y., *Land surface temperature retrieval from CBERS-02 thermal data and its application in quantitative analysis of UHI effect.* Journal of Remote Sensing, 2006. **10**(4): p. 789-797.
46. Al-Kafy, A., *Prediction of seasonal urban thermal field variance index using machine learning algorithms in Cumilla, Bangladesh.* Sustainable Cities and Society, 2021. **64**: p. 102542.
47. Mary C. Sheehan, F.K., Gurtejandeep S. Swaich, William Hines, Shivani Mehta, Mary A. Fox, *Thermal and ecological assessment based on land surface temperature in Bogura, Bangladesh.* PLOS Climate, 2021. **1**(5): p. e0000012.
48. Muñoz-Sabater, J. and et al., *ERA5-Land: a global high-resolution reanalysis dataset for land applications.* Earth System Science Data, 2021. **13**(9): p. 4349-4383.
49. Hersbach, H., et al., *The ERA5 global reanalysis.* Quarterly Journal of the Royal Meteorological Society, 2020. **146**(730): p. 1999-2049.
50. Hamann, C. and J. de Kadt, *Quality of Life Survey 6 (2020/21): Sample Design*, in *Gauteng City-Region Observatory (GCRO).* 2021.

51. Gauteng City-Region, O., *Quality of Life Survey V (2017/18): Data Report*, in *Gauteng City-Region Observatory (GCRO)*. 2019.
52. Heaviside, C., H. Macintyre, and S. Vardoulakis, *The Urban Heat Island: Implications for health in a changing environment.* Current Environmental Health Reports, 2017. **4**(3): p. 296-305.
53. Santamouris, M., *Innovative heat mitigation and the role of urban greenery.* Solar Energy, 2020. **204**: p. 682-699.
54. Chen, X., H. Zhao, and P. Li, *Remote sensing image-based analysis of the relationship between urban heat island and land use/cover changes.* Remote Sensing of Environment, 2006. **104**(2): p. 133-146.
55. Nori-Sarma, A. and et al., *Disparities in heat risk among urban populations.* The Lancet Planetary Health, 2022. **6**(1): p. e11-e13.
56. Watts, N., et al., *The 2023 report of The Lancet Countdown on health and climate change.* The Lancet, 2023. **402**(10499): p. 1614-1660.
57. Wright, C., M. Norval, and R. Hertle, *Socio-economic, infrastructural and health risk factors associated with adverse heat effects in South Africa.* The Pan African Medical Journal, 2019. **34**: p. 40.
58. Schuster, R.C., et al., *Heat vulnerability and socioeconomic status in urban areas.* Environmental Research, 2017. **154**: p. 304-313.
59. Ansah, E.W., Agyemang, S., & Nyarko, B. K. , *Heatwaves and health system access in Africa.* Global Health Action, 2024. **17**(1): p. 2182101.
60. Murage, P., Kovats, S., Sarran, C., Taylor, J., McInnes, R., & Hajat, S., *What factors increase the risk of heat-related mortality? A case-crossover study in London.* Environment International, 2020. **134**: p. 105292.
61. de Kadt, J., et al., *Quality of Life Survey 6 (2020/21): Overview Report*, in *Gauteng City-Region Observatory (GCRO)*. 2021.
62. Anselin, L., *Local Indicators of Spatial Association—LISA.* Geographical Analysis, 1995. **27**(2): p. 93-115.
63. Strauss, M., *A historical exposition of spatial injustice and segregated urban settlement in South Africa.* Fundamina, 2019. **25**(2): p. 135-168.

9. Supplementary materials

### 9.1. Table S1: Comparative Analysis of Key Socioeconomic and Healthcare Indicators Between GCRO Quality of Life Surveys (2017-2018 and 2020-2021)

| Indicator | 2017-2018 (%) | 2020-2021 (%) | Absolute Change (pp) | Relative Change (%) | Spatial Correlation | Rank Correlation |
|---|---|---|---|---|---|---|
| Unemployment | 3.76 | 18.67 | 14.91 | 396.5 | 0.044 (Very Weak) | 0.086 (Very Weak) |
| Food Insecurity | 7.13 | 61.69 | 54.56 | 765.2 | 0.582 (Moderate) | 0.612 (Moderate) |
| No Medical Insurance | 11.36 | 31.14 | 19.78 | 174.1 | 0.397 (Weak) | 0.440 (Weak) |
| Public Healthcare Use | 23.11 | 75.42 | 52.31 | 226.4 | 0.891 (Strong) | 0.760 (Strong) |
| Informal Dwellings | 62.57 | 11.23 | -51.34 | -82.1 | 0.815 (Strong) | 0.768 (Strong) |

Notes:

- pp = percentage points
- Spatial Correlation: Pearson correlation coefficient between ward-level values across surveys
- Rank Correlation: Spearman correlation coefficient measuring stability of ward rankings
- Interpretation of correlation strength: Very Weak (<0.2), Weak (0.2-0.4), Moderate (0.4-0.6), Strong (0.6-0.8), Very Strong (>0.8)
- Data source: GCRO Quality of Life Surveys 2017-2018 (v1.1) and 2020-2021 (new-weights-v1)

### 9.2. Table S2: Comparative Rankings of Combined Heat Vulnerability Index (HVI) and Component Factors

| Rank | Heat Vulnerability Index (HVI) Value Ward | Heat Vulnerability Index (HVI) Value Value | Heat Exposure Ward | Heat Exposure Value | Low Vegetation Ward | Low Vegetation Value | Public HC Ward | Public HC Value | No Insurance Ward | No Insurance Value | Hunger Risk Ward | Hunger Risk Value | Crowded Ward | Crowded Value |
|---|---|---|---|---|---|---|---|---|---|---|---|---|---|---|
| 0 | Ward 87 | 1 | Ward 116 | 30,66 | Ward 63 | 0,045 | Ward 128 | 98,6 | Ward 128 | 97,1 | Ward 2 | 70 | Ward 113 | 51,5 |
| 1 | Ward 117 | 0,97 | Ward 108 | 30,56 | Ward 116 | 0,054 | Ward 35 | 96,5 | Ward 61 | 94,5 | Ward 6 | 69 | Ward 95 | 43,4 |
| 2 | Ward 88 | 0,94 | Ward 113 | 30,37 | Ward 108 | 0,057 | Ward 127 | 95,6 | Ward 116 | 94,2 | Ward 48 | 66,7 | Ward 114 | 40,8 |

| | | | | | | | | | | | | | | |
|---|---|---|---|---|---|---|---|---|---|---|---|---|---|---|
| 3 | Ward 72 | 0,92 | Ward 135 | 30,37 | Ward 62 | 0,058 | Ward 21 | 94,8 | Ward 19 | 94,2 | Ward 121 | 64,4 | Ward 37 | 40,5 |
| 4 | Ward 90 | 0,92 | Ward 8 | 30,35 | Ward 133 | 0,065 | Ward 40 | 94,4 | Ward 111 | 92,9 | Ward 130 | 63,7 | Ward 107 | 40 |
| 5 | Ward 73 | 0,91 | Ward 107 | 30,14 | Ward 107 | 0,068 | Ward 24 | 93,5 | Ward 6 | 91,6 | Ward 15 | 63,2 | Ward 2 | 37,5 |
| 6 | Ward 103 | 0,9 | Ward 96 | 30,09 | Ward 77 | 0,075 | Ward 130 | 93,3 | Ward 75 | 91,6 | Ward 45 | 61,5 | Ward 75 | 37,2 |
| 7 | Ward 104 | 0,89 | Ward 32 | 30,06 | Ward 79 | 0,076 | Ward 50 | 93,3 | Ward 53 | 91,2 | Ward 47 | 61,3 | Ward 116 | 36 |
| 8 | Ward 99 | 0,88 | Ward 105 | 29,98 | Ward 76 | 0,076 | Ward 34 | 92,2 | Ward 59 | 91 | Ward 51 | 60,2 | Ward 35 | 35,7 |
| 9 | Ward 106 | 0,87 | Ward 76 | 29,88 | Ward 75 | 0,078 | Ward 11 | 92,1 | Ward 127 | 90,8 | Ward 41 | 60,1 | Ward 44 | 33,2 |

This table illustrates disparities between the Combined Heat Vulnerability Index (HVI) and its component factors (Heat Exposure, Vegetation, Healthcare Access, and Socio-Economic Conditions). Highlighting these differences underscores the multidimensional nature of heat vulnerability and the need for targeted interventions addressing both individual drivers and their combined impacts.

Supplementary Code and Data Repositories

To promote transparency and reproducibility, we have made all analysis code and data processing scripts publicly available in the following repositories:

1. GCRO Survey Comparison Analysis: https://github.com/Logic06183/GCRO
   - Contains scripts used to analyse and compare the 2017-2018 and 2020-2021 GCRO Quality of Life Surveys
   - Includes code for calculating correlations, generating visualisations, and quantifying pandemic impacts on key variables

2. Main Analysis Scripts and Data Sources: https://github.com/Logic06183/data_sources
   - Contains the complete analysis pipeline used in this study
   - Includes scripts for processing environmental data, calculating vulnerability indices, and performing spatial analyses
   - Contains visualisation code for generating all figures in the manuscript
   - Provides reproducible R scripts for Principal Component Analysis and LISA cluster analysis

We encourage interested researchers to use these resources for validation, extension, or application to other urban contexts.

### 9.3. Sensitivity Analysis for LST and UTFVI Variables

To address potential multicollinearity concerns between Land Surface Temperature (LST) and Urban Thermal Field Variance Index (UTFVI), we performed a sensitivity analysis examining how their inclusion or exclusion affects the Heat Vulnerability Index. The correlation between LST and UTFVI was very high (r = 0.995), confirming the need for this analysis.

We created three versions of the Heat Vulnerability Index:

1. Original: Including all 22 variables
2. No_LST: Excluding LST (21 variables)
3. No_UTFVI: Excluding UTFVI (21 variables)

Principal Component Analysis was performed for each version, and the results are summarized in Table S3.

**Table S3: Sensitivity Analysis Results for Different HVI Versions**

| Analysis Version | Variables | PC1 Variance (%) | Mean HVI | SD HVI |
|---|---|---|---|---|
| Original | 22 | 31.46 | 0.5606 | 0.2632 |
| No_LST | 21 | 31.75 | 0.5689 | 0.2777 |
| No_UTFVI | 21 | 32.02 | 0.5686 | 0.2758 |

The correlations between the three HVI versions were exceptionally high:

- Original vs. No_LST: r = 0.997
- Original vs. No_UTFVI: r = 0.997
- No_LST vs. No_UTFVI: r = 0.999

Most importantly, all three indices identified exactly the same wards in the top 10 most vulnerable areas, with only minor differences in their rankings. This high level of consistency demonstrates that our vulnerability assessment approach is robust to the inclusion or exclusion of either LST or UTFVI, despite their high correlation. These findings support the validity of our original analysis and indicate that multicollinearity between these two variables does not significantly impact the identification of vulnerability hotspots across Johannesburg.

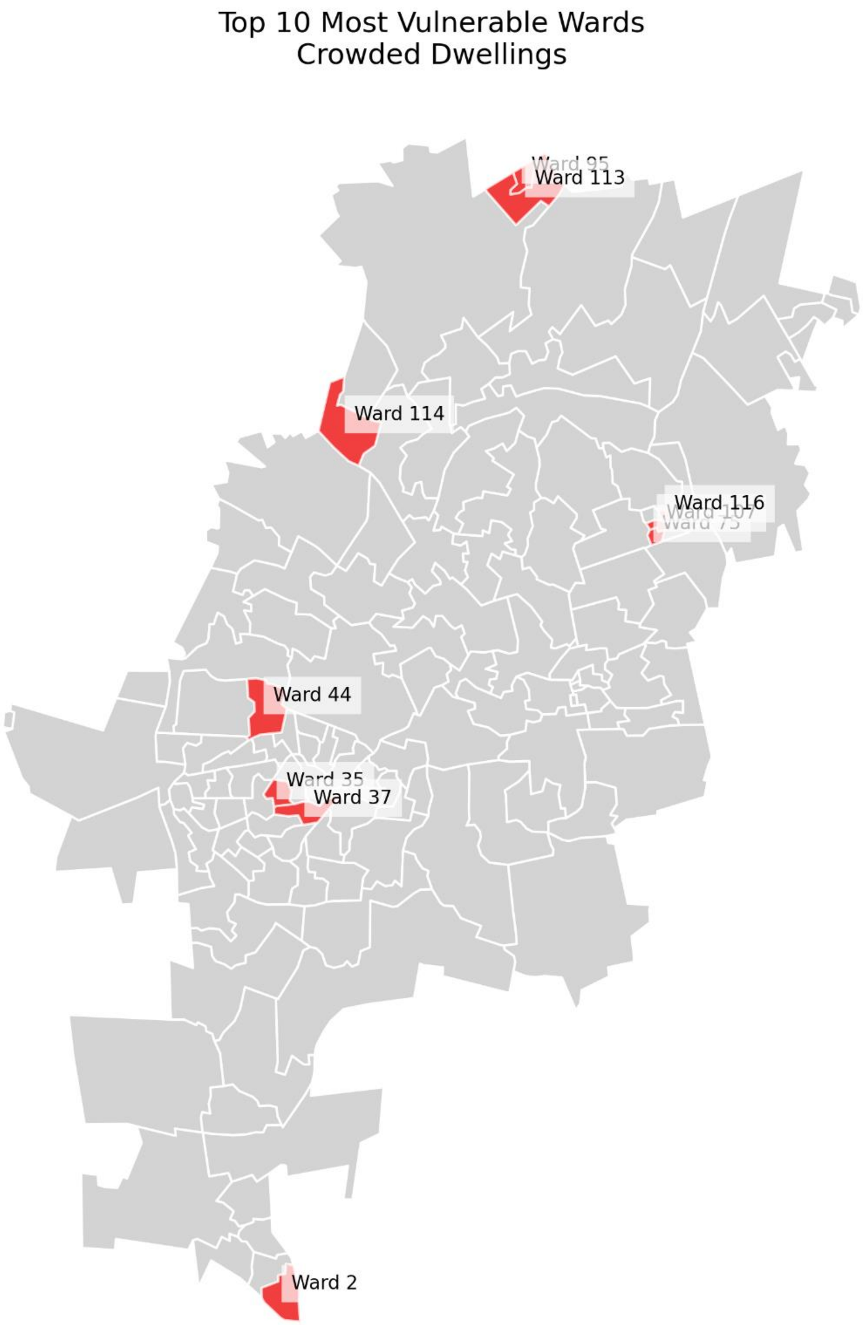

Top 10 Most Vulnerable Wards
Crowded Dwellings
Ward 95
Ward 113
Ward 114
Ward 116
Ward 107
Ward 44
Ward 35
Ward 37
Ward 2

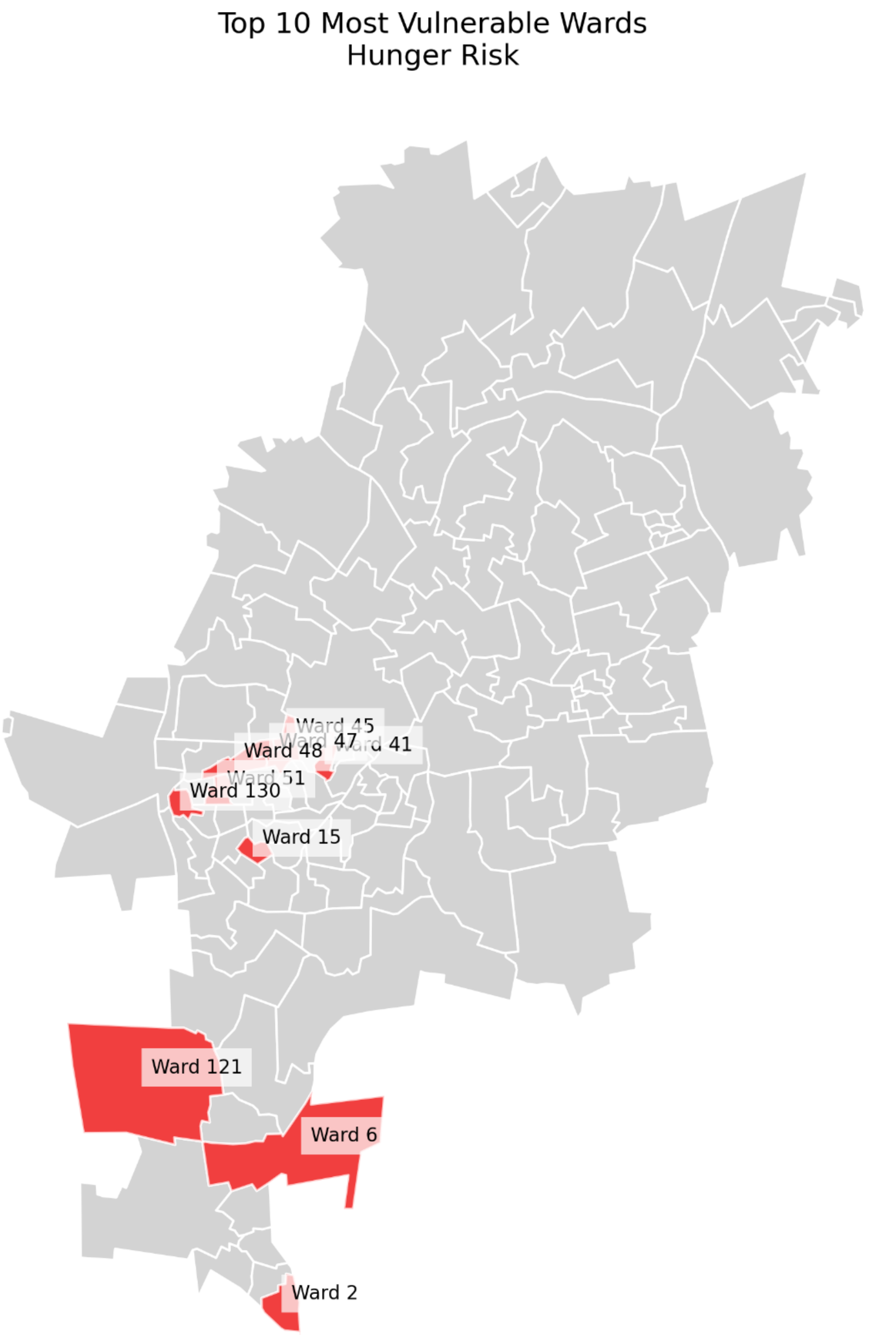

Top 10 Most Vulnerable Wards
Hunger Risk
Ward 45
Ward 47
Ward 41
Ward 48
Ward 51
Ward 130
Ward 15
Ward 121
Ward 6
Ward 2

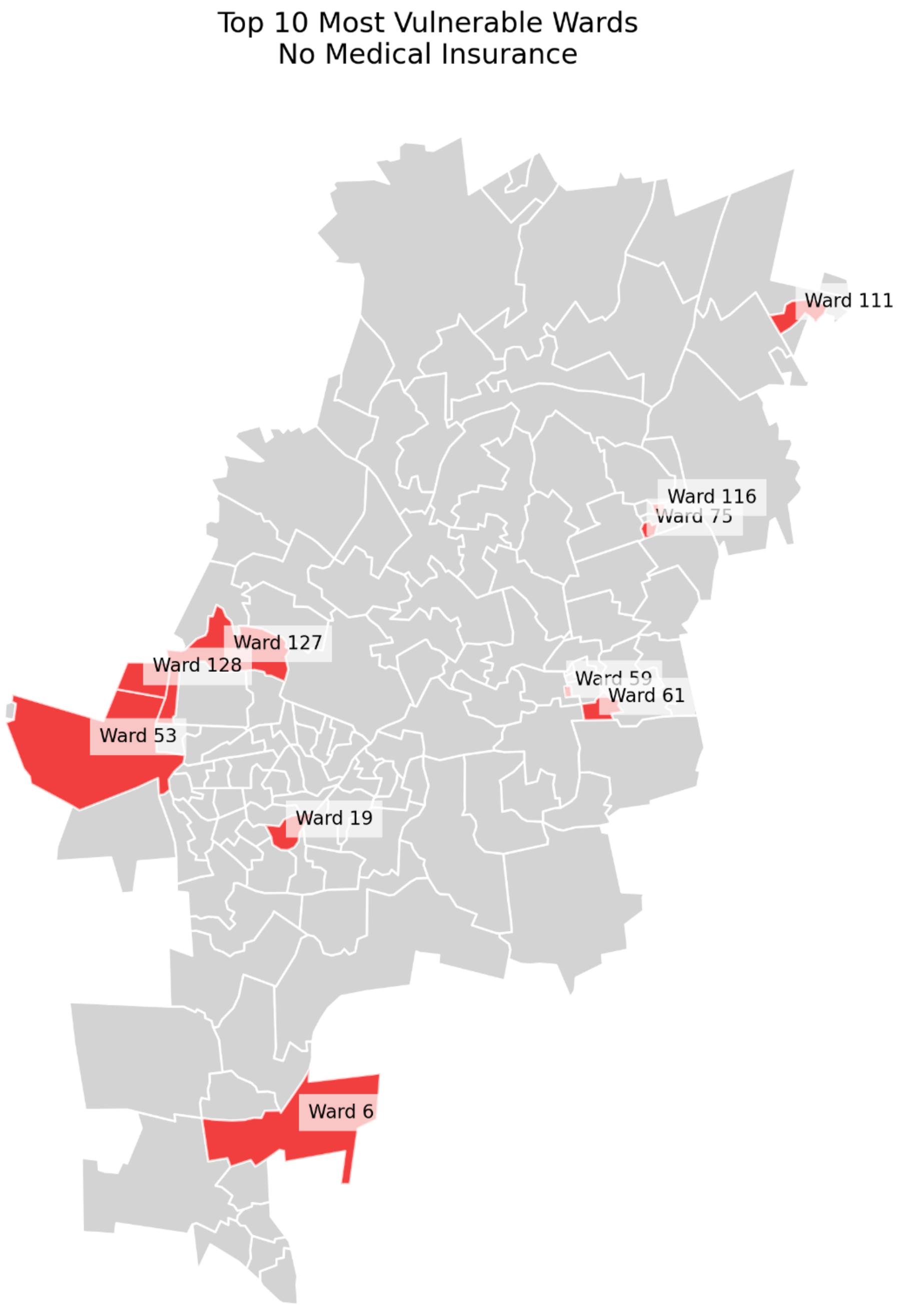

Top 10 Most Vulnerable Wards
No Medical Insurance
Ward 111
Ward 116
Ward 75
Ward 127
Ward 128
Ward 59
Ward 61
Ward 53
Ward 19
Ward 6

773

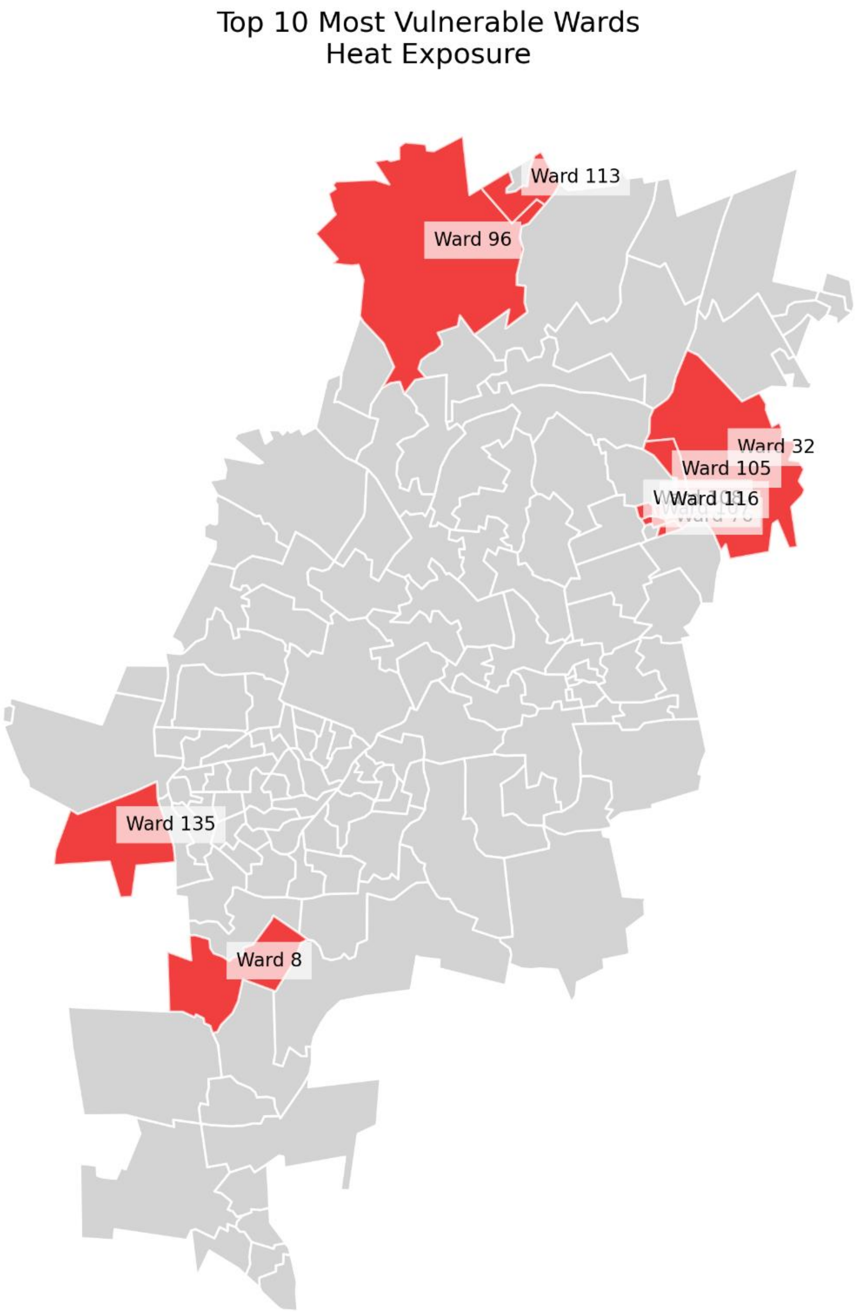

Top 10 Most Vulnerable Wards
Heat Exposure
Ward 113
Ward 96
Ward 32
Ward 105
Ward 116
Ward 135
Ward 8

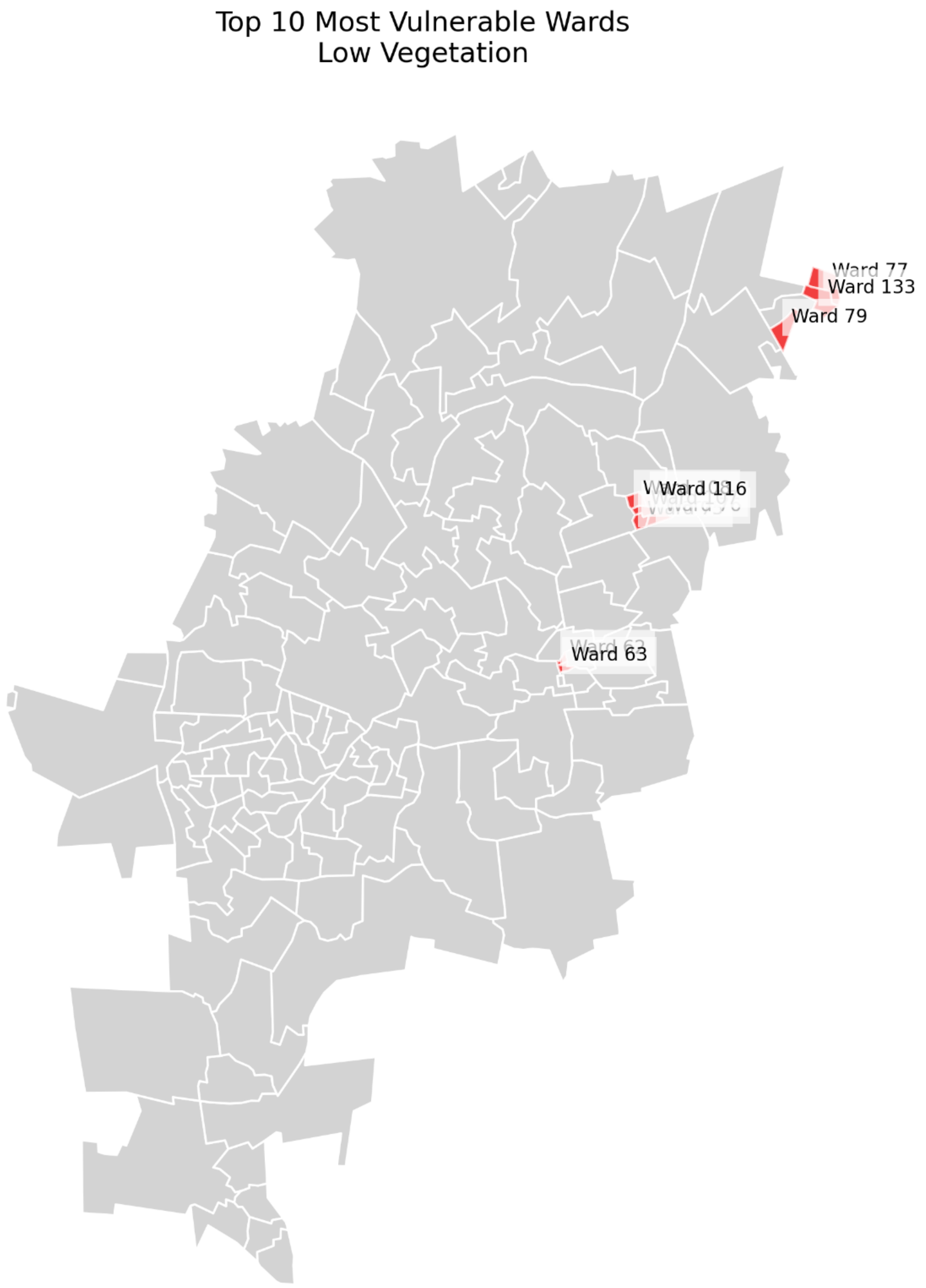

Top 10 Most Vulnerable Wards
Low Vegetation
Ward 77
Ward 133
Ward 79
Ward 116
Ward 63

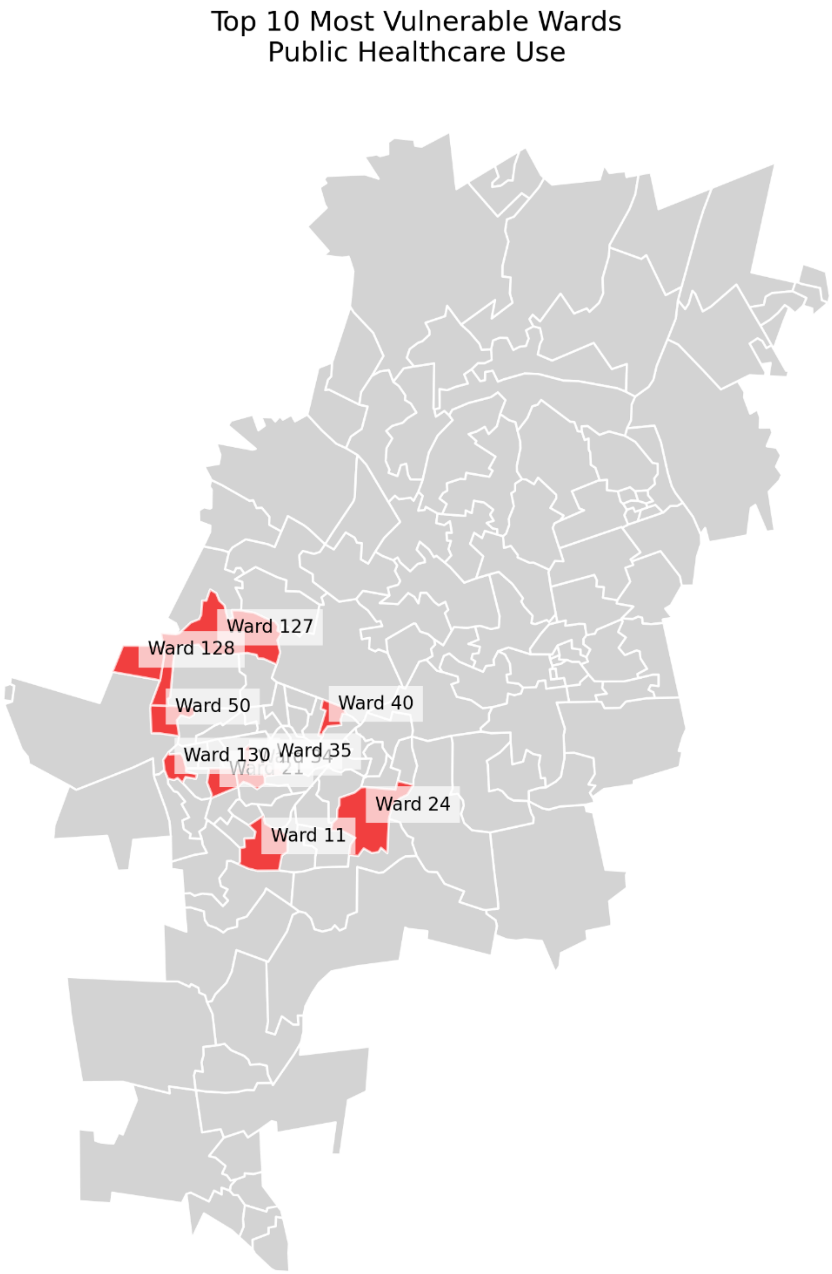

Top 10 Most Vulnerable Wards
Public Healthcare Use
Ward 127
Ward 128
Ward 50
Ward 40
Ward 130
Ward 35
Ward 21
Ward 24
Ward 11
